# Testing whether multi-level factors protect poly-victimised children against psychopathology in early adulthood: a longitudinal cohort study

**DOI:** 10.1017/S2045796024000660

**Published:** 2024-11-05

**Authors:** F. Blangis, L. Arseneault, A. Caspi, R. M. Latham, T. E. Moffitt, H. L. Fisher

**Affiliations:** 1 Social, Genetic & Developmental Psychiatry Centre, Institute of Psychiatry, Psychology & Neuroscience, King’s College London, London, UK; 2 Department of Psychology and Neuroscience, Duke University, Durham, NC, USA; 3 Department of Psychiatry and Behavioral Sciences, Duke University, Durham, NC, USA; 4 Duke University Population Research Institute, Duke University, Durham, NC, USA; 5 PROMENTA, Department of Psychology, University of Oslo, Oslo, Norway; 6 ESRC Centre for Society and Mental Health, King’s College London, London, UK

**Keywords:** adversity, child abuse, E-Risk cohort study, mental health, resilience

## Abstract

**Aims:**

Exposure to multiple forms of victimisation in childhood (often referred to as poly-victimisation) has lifelong adverse effects, including an elevated risk of early-adulthood psychopathology. However, not all poly-victimised children develop mental health difficulties and identifying what protects them could inform preventive interventions. The present study investigated whether individual-, family- and/or community-level factors were associated with lower levels of general psychopathology at age 18, among children exposed to poly-victimisation. Additionally, it examined whether these factors were specific to poly-victimised children or also associated with fewer mental health difficulties in young adults regardless of whether they had been poly-victimised.

**Methods:**

We used data from the Environmental Risk (E-Risk) Longitudinal Twin Study, a population-representative cohort of 2,232 children born in 1994–1995 across England and Wales and followed to 18 years of age (with 93% retention, n = 2,066). Poly-victimisation (i.e., exposure to two or more of physical abuse, sexual abuse, emotional abuse and neglect, physical neglect, bullying by peers, and domestic violence) and nine putative protective factors (intelligence quotient, executive functioning, temperament, maternal and sibling warmth, atmosphere at home, maternal monitoring, neighbourhood social cohesion, and presence of a supportive adult) were measured prospectively between ages 5 and 12 years from interviews with mothers and children, surveys of neighbours, child-protection referrals, and researchers’ observations. Early-adulthood psychopathology was assessed in interviews with each twin at age 18 and used to construct a latent factor of general psychopathology.

**Results:**

Approximately a third (n = 720) of participants were prospectively defined as exposed to poly-victimisation (53% male). Poly-victimised children had greater levels of general psychopathology at age 18 than non-poly-victimised children (adjusted [adj.] β = 4.80; 95% confidence interval [95% CI] 3.13, 6.47). Presence of a supportive adult was the only factor robustly associated with lower levels of general psychopathology among poly-victimised children (adj.β = −0.61; 95% CI −0.99, −0.23). However, this association was also evident in the whole sample regardless of poly-victimisation exposure (adj.β = −0.52; 95% CI −0.81, −0.24) and no significant interaction was observed between the presence of a supportive adult and poly-victimisation in relation to age-18 general psychopathology.

**Conclusions:**

Having at least one adult to turn to for support was found to be associated with less psychopathology in early adulthood among both poly-victimised and non-poly-victimised children. This suggests that strategies to promote better availability and utilisation of supportive adults should be implemented universally. However, it may be beneficial to target these interventions at poly-victimised children, given their higher burden of psychopathology in early adulthood.

## Introduction

Childhood victimisation (including physical abuse, sexual abuse, emotional abuse, neglect, exposure to domestic violence, and bullying by peers) can have lifelong adverse effects, with an elevated risk of psychopathology in early adulthood, especially when multiple forms of victimisation (or poly-victimisation) are experienced (Kessler *et al.*, 2010; Murphy *et al.*, 2020). Early adulthood is a critical developmental period to study as over 75% of adult mental health disorders have their onset by age 18 (Kim-Cohen *et al.*, 2003). Moreover, there is a high co-occurrence of psychopathology in early adulthood with nearly half of individuals with a mental disorder (comprising internalising, externalising and thought disorders) found to experience at least one additional mental disorder concurrently at age 21 (Newman *et al.*, 1996) and by mid-life 85% have been shown to experience more than one type of mental disorder (Caspi *et al.*, 2020). Therefore, it does not make sense to examine individual mental disorders. Furthermore, since victimisation exposure has been associated with nonspecific effects on multiple mental disorders (Daníelsdóttir *et al.*, 2024; Meehan *et al.*, 2020), its relationship with a general factor of psychopathology that captures the propensity to develop any type of mental disorder, also called ‘p’, is of interest (Caspi *et al.*, 2014; Schaefer *et al.*, 2018).

Although childhood victimisation significantly increases the risk for early-adulthood psychopathology, not all victimised children develop mental health difficulties in adulthood. For example, Meehan *et al.* (2020) found that 39.6% of victimised children within a British longitudinal cohort did not meet diagnostic criteria for any psychiatric disorder by age 18 years. Identifying factors that can protect such victimised children from developing early-adulthood psychopathology may help inform the content of targeted preventive mental health interventions. Protective factors for psychopathology are likely to be identified at various system levels (individual-, family- and community-level) and interact with each other, which is why the most effective interventions often focus on targeting protective factors across these levels (Ungar *et al.*, 2013; Ungar and Theron, 2020). At the individual level in childhood, these factors may include cognitive abilities (e.g., relatively high intelligence quotient [IQ], strong executive functioning), self-regulation (e.g., internal locus of control, an easy-going temperament, cognitive flexibility, ego-control), adaptive coping skills, and prosocial behaviour, all of which may enable children to seek help following victimisation (Nelson-Le Gall, 1981) and respond adaptively to these stressful experiences (Compas *et al.*, 2001), thus mitigating victimisation’s adverse effects on antisocial behaviour and adult mental disorders (Jaffee, 2017; Jaffee *et al.*, 2007; Yule *et al.*, 2019). At the family and community levels, protective factors may provide a safe environment where the child can seek help and find support to compensate for victimisation (Ozer *et al.*, 2017). Family-level characteristics that have been shown to be protective against emotional and behavioural problems and mental disorders in children exposed to various forms of victimisation encompass warmth from the mother towards the child, parental monitoring, family cohesion, living in a nurturing home environment, and the quality of siblings’ relationships with one another (Bowes *et al.*, 2010; Collishaw *et al.*, 2007; Yule *et al.*, 2019). Additionally, community-level characteristics, such as social cohesion within the surrounding neighbourhood, have been shown to protect victimised children from developing psychotic experiences and other psychopathology (Crush *et al.*, 2018a; Yule *et al.*, 2019). Lastly, access to socially supportive relationships within the family (e.g., parents and siblings) and in the wider community (e.g., friends, teachers, neighbours) have also been found to exert a protective influence against a wide range of mental health difficulties among children exposed to violence and other forms of victimisation within and outside of the home (Collishaw *et al.*, 2007; Crush *et al.*, 2018b; Jaffee, 2017; Latham *et al.*, 2022).

However, our understanding of factors associated with lower levels of psychopathology in early adulthood is limited, primarily because previous studies have often focused on specific mental disorders (Yule *et al.*, 2019). This narrow focus can reduce the ability to identify associations resulting from unaddressed similarities between interrelated disorders (Caspi *et al.*, 2024). It is also based on studies focusing on psychopathologies that are present in childhood or early adolescence, whose results may not be generalisable to psychopathology in early adulthood (Crush *et al.*, 2018a). Moreover, some research has focused on specific types of childhood victimisation that can either strengthen or weaken associations (Herrenkohl *et al.*, 2005) and this does not reflect real-world experiences where children are often exposed to more than one type of victimisation (Turner *et al.*, 2010). Others have investigated childhood poly-victimisation through retrospective self-reports (Afifi *et al.*, 2016), often capturing different groups of victimised children compared to those identified with prospective measures (Baldwin *et al.*, 2019).

To address these knowledge gaps, the present study utilises prospectively collected data from the Environmental Risk (E-Risk) Longitudinal Twin Study, a large, nationally representative cohort of children born in the UK. Potential protective factors were selected based on the existing literature, their ability to reflect three system levels within which children grow up (individual-, family-, community-level factors), as well as their availability within the E-Risk cohort, and their relevance for developing and targeting effective preventive interventions. Therefore, our aim was to investigate whether specific individual factors (IQ, executive functioning, approach temperament), family-related factors (maternal warmth, sibling warmth, atmosphere at home, maternal monitoring), community-related factors (neighbourhood social cohesion) and family- and community-related factors (presence of a supportive adult) were (i) associated with lower levels of general psychopathology at age 18 among poly-victimised children (i.e., were protective against poor mental health in this high-risk group) and (ii) also associated with fewer mental health difficulties in young adults regardless of whether they had or had not been poly-victimised (i.e., were more widely promotive of good mental health in the general population; Brumley and Jaffee, 2016).

## Methods

### Study cohort

Participants were members of the E-Risk Longitudinal Twin Study, which tracks the development of a nationally representative birth cohort of 2,232 twin children born in England and Wales in 1994–1995. Full details about the sample are reported elsewhere (Moffitt and E‐Risk Study Team, 2002) and in Supplementary Text 1. Briefly, the E-Risk sample was constructed in 1999–2000 when 1,116 families (93% of those eligible and of whom 90.4% were White) with same-sex 5-year-old twins participated in home-visit assessments. This sample comprised 56% monozygotic and 44% dizygotic twin pairs; sex was evenly distributed within zygosity (49% male). Families were recruited to represent the UK population of families with newborns in the 1990s, on the basis of residential location throughout England and Wales and mother’s age.

Follow-up home-visits were conducted when children were aged 7, 10, 12 and 18 (participation rates were 98%, 96%, 96% and 93%, respectively). At age 18, a total of 2,066 participants were assessed. Average age at time of assessment was 18.4 years (standard deviation [SD] = 0.36); all interviews were conducted after the 18th birthday. There were no differences between those who did and did not take part at age 18 in terms of socioeconomic status (SES) assessed when the cohort was initially defined (χ^2^ = 0.86, *p* = .65), age-5 IQ scores (t = 0.98, *p* = .33), age-5 internalising or externalising behaviour problems (t = 0.40, *p* = .69 and t = 0.41, *p* = .68, respectively) or childhood poly-victimisation (z = 0.51, *p* = .61). The Joint South London and Maudsley and Institute of Psychiatry Research Ethics Committee approved each study phase. Parents gave informed consent and twins gave assent between 5 and 12 years and then informed consent at age 18. This study followed the Strengthening the Reporting of Observational Studies in Epidemiology (STROBE) guidelines (von Elm *et al.*, 2007).

### Measures

#### Childhood victimisation

Prospective measures of victimisation utilised in this cohort and the coding criteria are described elsewhere (Danese *et al.*, 2017; Fisher *et al.*, 2015) and in Supplementary Text 2. In brief, lifetime exposure to several types of victimisation was assessed repeatedly when children were 5, 7, 10 and 12 years. Comprehensive dossiers were compiled for each child with cumulative information about: exposure to domestic violence between mother and partner; frequent bullying by peers; physical abuse by an adult; sexual abuse; emotional abuse and neglect; and physical neglect, between birth and age 12. Dossiers comprised reports from caregivers, recorded narratives of caregiver interviews, recorded debriefings with research workers who had coded any indications of abuse and neglect at any of the successive home visits, interviews with children about their bullying experiences, and information from clinicians whenever the study team made a child-protection referral. These were reviewed by two independent researchers and rated for the presence and severity (none/mild/severe) of each type of victimisation. For example, children in families in which no physical violence took place were coded as not having been exposed to domestic violence; children in families in which physical violence took place on one occasion were coded as having been exposed to mild domestic violence; and children in families in which physical violence took place on multiple occasions were coded as having been exposed to severe domestic violence. How the severity of each type of victimisation was defined is provided in Supplementary Text 2. Poly-victimisation was defined as experiencing two or more types of mild or severe victimisation before age 12 (N = 720, 35%) compared to one or none (N = 1,346, 65%).

For sensitivity analyses, we used victimisation measured retrospectively using the Childhood Trauma Questionnaire when participants were aged 18 (Bernstein and Fink, 1998; Newbury *et al.*, 2018) (Supplementary Text 2). Participants reported on their personal experiences of physical, sexual and emotional abuse, and physical and emotional neglect, for the period before they were aged 12. For comparability to the prospective measure of poly-victimisation, we added domestic violence and bullying by peers from the prospective report to the ‘self-reported victimisation’ variable. Retrospective poly-victimisation was defined as experiencing two or more types of moderate or severe victimisation before age 12 (N = 556, 27%) compared to none or mild victimisation (N = 1,510, 73%).

#### Early-adulthood psychopathology

We utilised a continuous latent factor of general psychopathology, also known as ‘p’, derived using a confirmatory factor analysis by fitting a bi-factor model to 11 symptom scales (post-traumatic stress disorder, major depressive disorder, generalised anxiety disorder, disordered eating, attention-deficit hyperactivity disorder, conduct disorder, alcohol dependence, cannabis dependence, nicotine dependence, psychotic symptoms, and prodromal symptoms) obtained from data collected during private interviews with each twin at age 18 about psychopathology in the previous year (Schaefer *et al.*, 2018) (see Supplementary Text 3 and Supplementary Figure S1). For sensitivity analyses, we also utilised the three specific underlying dimensions of psychopathology (internalising symptoms, externalising symptoms, and thought disorder symptoms) derived from the bi-factor model (see Supplementary Text 3 and Supplementary Figure S1). All scores were scaled to a mean of 100 and SD of 15.

#### Putative protective factors

Table 1 provides information on the measures, sources, and age at which individual factors (IQ, executive functioning, approach temperament), family-related factors (maternal warmth, sibling warmth, atmosphere at home, maternal monitoring), community-related factors (neighbourhood social cohesion) and family- and community-related factors (presence of a supportive adult) were obtained.

**Table 1.** Description of the putative protective factors analysed in this study

Measure	Respondent	Description of the measure	Age at evaluation, years
*Individual-level protective factors*			
IQ	Child	Two subtests of the Wechsler Preschool and Primary Scale of Intelligence (WPPSI) Revised (Wechsler, 1990) were administered to children: Vocabulary and Block Design. IQ scores were prorated following procedures described by Sattler (Sattler, 1992) and then standardised with a mean of 100 and standard deviation of 15.	5
Executive functioning	Child	Executive function was measured as the mean score of three separate tasks administered to children: Mazes (Grodzinsky and Diamond, 1992), a WPPSI subtest; Day-Night (Gerstadt *et al.*, 1994), a nonverbal analogue of the Stroop task; and Sentence Working Memory, based on the Baddeley model of working memory (Baddeley, 1986; Baddeley, 1996), after converting each scale to a common metric. The resulting combined score was standardised with a mean of 100 and standard deviation of 15.	5
Temperament (approach)	Interviewer	Research workers rated each twin on 25 different behavioural characteristics that assessed children’s style of approach and response to the testing session. The behavioural characteristics were derived from scales initially used to rate children enrolled in the American Collaborative Study on Cerebral Palsy, Mental Retardation, and Other Neurological Disorders of Infancy and Childhood (Goldsmith and Gottesman, 1981) and were modified for use in the Dunedin Health and Development Study (Caspi *et al.*, 1995; Henry, 1999). The current study used the measure for ‘Approach’ made up of six items including quick adjustment, friendliness, self-confidence, talkativeness, easy separation, and smiling and laughter (internal consistency: α = 0.90).	5
*Family-level protective factors*			
Maternal warmth	Mother, coded by independent raters	Assessed using procedures adapted from the Five-Minute Speech Sample method (Magaña *et al.*, 1986). Mothers were asked to speak for 5 minutes about each of their children when they were aged 5 and again at age 10. Warmth was coded on a six-point scale from no warmth (complete absence of warmth) to high warmth (definite warmth, enthusiasm, interest in, and enjoyment of the child). Two trained raters, blind to all other E-Risk Study data, coded the tapes of the mothers’ speech sample (inter-rater agreement: r = 0.90) (Caspi *et al.*, 2004).	5 and 10 combined^a^ (significantly correlated, r = 0.37; *p*< .001)
Sibling warmth	Mother	Mothers were asked a series of questions about the quality of their children’s relationship with one another when the children were aged 7 and 10 (Jaffee *et al.*, 2007). Mothers responded on a three-point scale to six questions (e.g., ‘do your twins love each other’, ‘do both your twins do nice things for each other’). The internal consistency reliability score at age 7 was 0.77 and at age 10 was 0.80.	7 and 10 combined^a^ (significantly correlated, r = 0.57; *p*< .001)
Atmosphere at home	Interviewer	Derived from the Coder’s Impression Inventory, which is based on the Home Observation for Measurement of the Environment (Bradley and Caldwell, 1977) and the University of Washington Parenting Clinic Questionnaire (Parent–Child Observations) (Webster-Stratton, 1998). The Coder’s Impression Inventory was rated immediately following the study visit at ages 7 and 10 by interviewers who had undergone 4-day training. This measure comprised items representing the state of the home (e.g., ‘Are visible rooms of the house clean?’), stimulation (e.g., ‘Is the children’s art displayed in the home?’), happiness (e.g., ‘Is this a happy home?’) and chaos (e.g., ‘Is the house chaotic or overly noisy?’). The internal consistency at age 7 was α = 0.77 and α = 0.79 at age 10.	7 and 10 combined^a^ (significantly correlated, r = 0.64; *p*< .001)
Maternal monitoring	Mother	Report on how closely the mother monitors her child’s behaviour when the child is away from home. Ten items were adapted from the Monitoring and Supervision Questionnaire (internal consistency: α = 0.63) (Stattin and Kerr, 2000) to capture whether the mother knew the friends the child hangs out with, where they go in their spare time, how they spend their money, what type of homework the child has, when the child has tests or projects, and how the child performs in different subjects. Mothers also reported on whether the child needs permission to leave home, needs permission before deciding what to do on the weekend, reports on where and who they are going out with, and reports on what they did when they return home.	10 and 12 combined^a^ (significantly correlated, r = 0.37; *p*< .001)
*Community-level protective factors*			
Neighbourhood social cohesion	Residents living alongside E-Risk families	Assessed via a postal survey. Survey respondents, who were typically living on the same street or within the same apartment block as the Study participants, reported on various characteristics of their immediate neighbourhood, including levels of neighbourhood social cohesion. Surveys were returned by an average of 5.18 (SD = 2.73) respondents per neighbourhood, and there were at least 2 responses for 95% of neighbourhoods (N = 5,601 respondents). Neighbourhood social cohesion was represented by five items (Sampson *et al.*, 1997). Respondents were asked how strongly they agreed (coded from 4 = strongly agree to 0 = strongly disagree) that ‘people around here are willing to help their neighbours’, ‘this is a close-knit neighbourhood’, ‘people in this neighbourhood can be trusted’, ‘people in this neighbourhood generally don’t get along with each other’ (reverse scored), and ‘people in this neighbourhood do not share the same values’ (reverse scored). Scores for each E-Risk family were then created by averaging the summary scores of respondents within that family’s neighbourhood.	13−14
*Protective factors that cross the family and community levels*			
Presence of a supportive adult	Child	The child was asked questions about whether they had a stable adult figure to rely on for basic needs and support (e.g., ‘there is an adult who I can tell almost anything to’, ‘there is an adult who I can go to if I am in trouble’). We derived a total score by summing responses to the 13 items (internal consistency: α = 0.85) (Crush *et al.*, 2018a). The questions did not ask the child to specify who the adult was, and thus, this could have been someone within or outside of their family.	12

E-Risk, Environmental Risk Longitudinal Twin Study; IQ, intelligence quotient.

a Averaged to provide a single score. In the absence of one measure, the available score was utilised.

#### Confounders

The biological sex of the child was reported by mothers at birth. Family SES was measured via a composite of total household income, highest maternal/paternal education and highest maternal/paternal occupation when children were aged 5. These three indicators were highly correlated (r values ranged from 0.57 to 0.67, all *p* values < .05) and loaded significantly onto one latent factor (factor loadings = 0.80, 0.70 and 0.83 for income, education and occupation, respectively). This latent variable was then categorised into tertiles (i.e., low-, medium- and high-SES) (Trzesniewski *et al.*, 2006). In private interviews when the children were aged 12, mothers reported on family history of DSM disorders (Weissman *et al.*, 2000), which was converted to a proportion (0–1.0) of family members with a history of psychiatric disorders (Milne *et al.*, 2008).

### Statistical analyses

The premise and analysis plan for this project were preregistered at https://sites.duke.edu/moffittcaspiprojects/files/2023/12/Blangis_2023_Protective-factors-and-psychopathology.pdf. We conducted multiple linear regression analyses within STATA 16.1 (StataCorp, College Station, TX, USA) and accounted for the non-independence of our twin observations in all analyses using the Huber–White variance estimator (Rogers, 1993). First, we tested associations between the presence/absence of childhood poly-victimisation and levels of general psychopathology at age 18 in the whole sample to establish whether poly-victimised children had elevated psychopathology in early adulthood compared to their non-poly-victimised peers. Second, we investigated the associations between each putative protective factor and levels of general psychopathology at age 18 within the subsample of poly-victimised children to examine if any of these exerted a protective effect in this high-risk group. We utilised standardised beta coefficients to compare the relative impact of each factor on general psychopathology. Third, in the whole sample, we tested interactions between childhood poly-victimisation and the putative protective factors found to be significantly associated with lower levels of general psychopathology in step 2, and their associations with general psychopathology at age 18. This tested whether these factors were associated with reduced psychopathology only in poly-victimised children (significant interaction) or also in non-poly-victimised children (no interaction and evidence of a main association) and thus might be exerting a promotive effect in the whole sample. All these analyses were subsequently adjusted for biological sex, family SES, and family psychiatric history to take into account these potentially confounding factors. The precision of the estimated associations was determined using 95% confidence intervals (CIs) and those that did not include zero were considered to indicate statistical significance at *p*< .05.

Additionally, we conducted sensitivity analyses by repeating the first two steps, limiting the analyses to the factors found to be significantly associated with lower general psychopathology in the main analyses: (i) separately for each of the three domains of early-adulthood psychopathology (internalising, externalising and thought disorder symptom dimensions); (ii) using retrospective assessments of childhood maltreatment obtained at age 18 to define which children had experienced poly-victimisation; and (iii) defining poly-victimisation using only types of victimisation rated as severe. The latter two sensitivity analyses were conducted with general psychopathology as the outcome and then with the three specific domains of early-adulthood psychopathology as the outcomes. Analyses reported here were checked for reproducibility by an independent data-analyst, who recreated the code by working from the manuscript and applied it to a fresh dataset.

## Results

The characteristics of children included in the analysis (N = 2,066) are provided in Table 2, for the sample overall and separately for children who were and were not poly-victimised. Approximately a third (n = 720) of twin participants were prospectively defined as exposed to poly-victimisation (53.3% male). The most common forms of victimisation among poly-victimised children were exposure to domestic violence (86.3%; n = 621) and being bullied by peers (78.2%; n = 563). Among the prospectively defined poly-victimised children, 71.8% (n = 517) retrospectively reported having experienced poly-victimisation. Just over half of the prospectively defined poly-victimised children grew up in low SES families (52.4%), and on average they had a greater proportion of family members with a psychiatric history and slightly higher mean psychopathology scores than non-poly-victimised children (Table 2).

**Table 2.** Characteristics of children in the whole sample and separately for children who were and were not poly-victimised

	Whole sample (N = 2,066)		Not poly-victimised (N = 1,346)		Poly-victimised (N = 720)	
Characteristics	N	n (%), median [IQR], or mean (SD)	N	n (%), median [IQR], or mean (SD)	N	n (%), median [IQR], or mean (SD)
**Biological sex**	2,066		1,346		720	
Girls		1,085 (52.5%)		597 (44.4%)		336 (46.7%)
Boys		981 (47.5%)		749 (55.7%)		384 (53.3%)
**Family socioeconomic status**	2,066		1,346		720	
Low		691 (33.5%)		314 (23.3%)		377 (52.4%)
Medium		684 (33.1%)		483 (35.9%)		201 (27.9%)
High		691 (33.5%)		549 (40.8%)		142 (19.7%)
**Family psychiatric history**	2,010	0.4 (0.3)	1,304	0.3 (0.2)	706	0.5 (0.3)
**General psychopathology**	2,066	100 (15)	1,346	97.6 (14.1)	720	104.4 (15.7)
**Internalising disorders**	2,066	100 (15)	1,346	97.8 (14.2)	720	104.1 (15.7)
**Externalising disorders**	2,066	100 (15)	1,346	97.6 (14.1)	720	104.4 (15.6)
**Thoughts disorders**	2,066	100 (15)	1,346	97.8 (13.9)	720	104.2 (16.0)
**Victimisation**						
Exposure to domestic violence	2,066	937 (45.4%)	1,346	316 (23.5%)	720	621 (86.3%)
Bullying by peers	2,062	924 (44.8%)	1,342	361 (26.9%)	720	563 (78.2%)
Physical abuse	2,066	415 (20.1%)	1,346	46 (3.4%)	720	369 (51.3%)
Sexual abuse	2,066	33 (1.6%)	1,346	3 (0.2%)	720	30 (4.2%)
Emotional abuse/neglect	2,066	240 (11.6%)	1,346	4 (0.3%)	720	236 (32.8%)
Physical neglect	2,066	185 (9.0%)	1,346	10 (0.7%)	720	175 (24.3%)
**Poly-victimisation retrospectively reported**	2,066	556 (26.9%)	1,346	39 (2.9%)	720	517 (71.8%)
**Protective factors**						
IQ	2,052	95.9 (14.6)	1,339	97.9 (14.2)	713	92.2 (14.6)
Executive function	2,051	11.6 (3.1)	1,341	11.8 (3.0)	710	11.3 (3.1)
Temperament (approach)	2,061	10 [8, 12]	1,344	11 [8, 12]	717	10 [7, 12]
Maternal warmth	2,056	3.5 (0.8)	1,337	3.6 (0.8)	719	3.3 (0.9)
Sibling warmth	2,052	10 [9, 12]	1,335	11 [10, 12]	717	10 [9, 11]
Atmosphere at home	2,050	28 [24, 30]	1,333	29 [25, 31]	717	25 [19, 28]
Maternal monitoring	2,044	19 [18, 20]	1,326	20 [18, 20]	718	19 [17, 20]
Neighbourhood social cohesion	1,994	2.2 (0.5)	1,295	2.3 (0.5)	699	2.1 (0.5)
Supportive adult	1,999	25 [23, 26]	1,301	25 [23, 26]	698	25 [22, 26]

IQ, intelligence quotient; IQR, interquartile range; SD, standard deviation. Percentages may not total 100 due to rounding.

### Is poly-victimisation in childhood associated with early-adulthood psychopathology?

Poly-victimised children had greater levels of general psychopathology at age 18 (mean = 104.4; SD = 15.7) than non-poly-victimised children (mean = 97.6; SD = 14.1) (unadjusted β = 6.74; 95% CI 5.19, 8.30). The association was slightly attenuated but remained robust after adjusting for the child’s biological sex, family SES, and family history of mental health disorders (adjusted β = 4.80; 95% CI 3.13, 6.47).

### Are individual-, family- or community-level factors associated with lower levels of general psychopathology among poly-victimised children?

Associations between each putative protective factor and general psychopathology at age 18 within the sub-group of poly-victimised children (N = 720) are presented in Table 3. Presence of a supportive adult was the only factor that demonstrated a robust association with lower levels of general psychopathology among poly-victimised children (adjusted β = −0.61; 95% CI −0.99, −0.23). Compared to the other factors, presence of a supportive adult had the strongest effect (standardised β = −0.15). Small protective effects were observed for a positive atmosphere at home (standardised β = −0.08), higher maternal monitoring (standardised β = −0.05), and greater neighbourhood social cohesion (standardised β = −0.06), but these associations were not statistically significant after accounting for potential confounders, and the relatively wide 95% CIs for social cohesion indicated this estimate was particularly imprecise. None of the individual factors were found to be protective in this poly-victimised group.

**Table 3.** Associations between individual-, family- and community-level factors and general psychopathology among poly-victimised children

	General psychopathology		
Protective factors^a^	β (95% CI) unadjusted	β (95% CI) adjusted^b^	Standardised β adjusted^b^
**IQ**	−0.04 [−0.13, 0.04]	−0.02 [−0.12, 0.08]	−0.02
**Executive function**	−0.08 [−0.47, 0.31]	−0.04 [−0.44, 0.35]	−0.01
**Temperament (approach)**	0.12 [−0.29, 0.53]	0.25 [−0.16, 0.66]	0.05
**Maternal warmth**	0.01 [−1.49, 1.52]	0.30 [−1.22, 1.81]	0.02
**Sibling warmth**	−0.33 [−1.09, 0.43]	−0.15 [−0.92, 0.63]	−0.02
**Atmosphere at home**	**−0.24 [−0.42, −0.06]**	−0.19 [−0.39, 0.01]	−0.08
**Maternal monitoring**	−0.60 [−1.20, −0.00]	−0.41 [−1.04, 0.23]	−0.05
**Neighbourhood social cohesion**	−2.47 [−5.20, 0.26]	−1.72 [−4.59, 1.15]	−0.06
**Supportive adult**	**−0.65 [−1.02, −0.28]**	**−0.61 [−0.99, −0.23]**	−0.15

CI, confidence interval; IQ, intelligence quotient.

All analyses account for the non-independence of twin observations.

The n varied from n = 685 to n = 719, due to different levels of completion of the measures.

Bold text indicates 95% CIs that do not include zero.

a Each factor was tested in separate linear regression models.

b Adjusted for biological sex, family socioeconomic status, and family history of psychiatric disorders.

### Are these factors specific to poly-victimised children?

Within the whole sample (including those who were and were not exposed to poly-victimisation), the presence of a supportive adult was associated with lower levels of general psychopathology (unadjusted β = −0.54; 95% CI −0.82, −0.25) even following adjustment for potential confounders (adjusted β = −0.52; 95% CI −0.81, −0.24). The protective influence of this factor on psychopathology was not specific to poly-victimised children given that no significant interaction was observed between poly-victimisation and the presence of a supportive adult in the whole sample (unadjusted β interaction = −0.11; 95% CI −0.57, 0.35; and adjusted β interaction = −0.09; 95% CI −0.56, 0.38).

### Sensitivity analyses

First, consistent patterns of associations were observed when dividing early-adulthood psychopathology into internalising, externalising and thought disorder symptom dimensions (Supplementary Tables S1 and S2).

Second, similar results were observed when childhood victimisation was (partially) measured retrospectively at age 18 (Supplementary Tables S1, S3 and S4).

Third, when restricting exposure to two or more types of severe victimisation in childhood, associations of a similar magnitude were observed although these were non-significant (Supplementary Tables S1, S5 and S6).

## Discussion

In this study, we investigated individual-, family- and community-level putative protective factors between childhood poly-victimisation and early-adulthood psychopathology. We found that the presence of a supportive adult at age 12 was significantly associated with lower levels of general psychopathology in early adulthood among poly-victimised children and also in young adults regardless of whether they had or had not been poly-victimised. Additionally, there was weak evidence from the adjusted standardised beta coefficients that a positive atmosphere at home, greater maternal monitoring, and higher neighbourhood social cohesion were associated with lower levels of general psychopathology in poly-victimised children, but the 95% CIs for these included zero and were therefore not statistically significantly. No individual factors were found to be associated with less early-adulthood general psychopathology. Sensitivity analyses investigating psychopathology split into its three main domains, childhood victimisation measured (partially) retrospectively at age 18, and when restricting to children exposed only to severe poly-victimisation, yielded associations of similar magnitudes. This suggests that the protective effects found may occur across the spectrum of mental health difficulties (rather than being specific to particular types of mental health difficulties) and largely hold regardless of whether prospective or retrospective reporting methods are utilised and the severity of victimisation experienced. It is important to note though that the associations for severe poly-victimisation were not statistically significant, which may have been due to the small size of this subgroup.

The main finding of this study was the robust promotive effects on mental health observed for the presence of a supportive adult. This finding aligns with previous research, suggesting that having at least one adult to whom children can turn is important in reducing the development of mental health difficulties, particularly for poly-victimised children (Jaffee, 2017; Jaffee *et al.*, 2007; Yule *et al.*, 2019). Studies have suggested that social support may have a stress-buffering effect, particularly by influencing the child’s biological responses to toxic stress (e.g., victimisation), or a direct effect on mental health through the development of secure and trusting relationships (Bauer *et al.*, 2021; Cohen and Wills, 1985; Jackson and Deye, 2015). The promotive effect of having a supportive adult was present regardless of poly-victimisation during childhood, consistent with existing literature (Evans *et al.*, 2013; McLewin and Muller, 2006). More research is therefore needed on the promotive effects on mental health of having a supportive adult in childhood among individuals within the general population who have not been exposed to specific risk factors.

Based on the effect sizes, we found small, but not statistically significant, protective effects for a positive atmosphere at home, greater maternal monitoring, and higher neighbourhood social cohesion factors. This is partially consistent with previous research that demonstrated how a caring and nurturing home environment can help a child who has experienced poly-victimisation to adjust, leading to improved mental health outcomes (Egeland *et al.*, 1993). Additionally, higher parental monitoring has been proposed to make a child feel looked after and supported (Ceballo *et al.*, 2003) and has been associated with lower levels of internalising and externalising problems (Kerr and Stattin, 2000), including among those exposed to violence (Bacchini *et al.*, 2011), though findings related to antisocial behaviour are mixed (Wertz *et al.*, 2016). Furthermore, living in a socially cohesive neighbourhood may provide opportunities for children to turn to those outside of their home for support following victimisation and promote better mental health (Leventhal and Brooks-Gunn, 2000). Future studies should explore the potentially protective effects of these factors among larger numbers of poly-victimised children.

### Strengths and limitations

Our study has several strengths. First, we utilised a nationally representative cohort study which allowed the collection of prospective measures of poly-victimisation, putative protective factors and mental health. Second, unlike other studies on this topic (Yule *et al.*, 2019), we defined psychopathology as a general factor as well as three dimensions of mental health symptoms, allowing for greater generalisability of our findings. Third, poly-victimisation was assessed both prospectively and retrospectively, allowing for a more comprehensive exploration of the associations using different measures of poly-victimisation (Baldwin *et al.*, 2019). Fourth, we conducted several sensitivity analyses that provided some reassurance about the consistency of the findings.

Our study also has several limitations. First, we assessed poly-victimisation during childhood without precise timing of exposure. Studies have shown that the impact of childhood adversity on mental health varies according to sensitive periods (McLaughlin, 2016; Murphy *et al.*, 2020). Knowing more about the temporality of each form of victimisation would also be beneficial for proposing targeted preventive interventions based on the age at which victimisation occurs. Second, we were not able to identify who the supportive adult was. Knowing whether the adult was a family member, someone from outside the family, from the neighbourhood or associated with the school, could provide valuable insights to inform preventive interventions, as the strength of the associations may differ depending on these factors. The promotive effect of the presence of a supportive adult may also vary according to the children’s state of mind, their perception of support, self-image, and social network (Bauer *et al.*, 2021). A study that is able to adjust for these variables and provides a more quantitative measure of the children’s social network, such as the number of sources of support, would allow for a more precise evaluation of the effect of having a supportive adult (Butler *et al.*, 2022). Third, we assessed the role of maternal warmth and monitoring but did not evaluate these factors in relation to fathers. Including these aspects of parenting from fathers in future studies would provide more comprehensive information. Fourth, we constructed the retrospective measure of poly-victimisation by including domestic violence and peer bullying from the prospective reports, as they were missing from the retrospective measures. This adjustment could have inadvertently strengthened the associations, especially given the high prevalence of domestic violence and bullying in the prospective reports. In addition, we included mild victimisation in the definition of poly-victimisation in the prospective measure, but not in the retrospective measures of maltreatment. This may have weakened the associations found with the retrospective measures though prevalence rates were similar. Fifth, we were unable to examine interactions with biological sex and SES due to insufficient power. Future studies using larger samples should consider including these interactions. Sixth, we did not assess whether the children in this sample had received any interventions, such as therapy, or consider a wider range of potential confounding factors, such as genetics, that may have influenced the association between poly-victimisation and psychopathology. Seventh, protective factors may vary over time and place, for example, according to societal and political developments (Ungar *et al.*, 2023). Our participants were born in the mid-1990s and all assessments were conducted before the COVID-19 pandemic, which has exacerbated psychological distress (Shanahan *et al.*, 2022). Further research is needed to validate our findings in current contexts. Finally, our results are derived from a cohort of predominantly White twins and the results may differ in the singleton population or in other ethnic groups. Although our findings have limited generalisability to ethnic minority groups within the UK, the E-Risk study population is representative of UK families in terms of geographical and socioeconomic distribution (Caspi *et al.*, 2000; Moffitt and E‐Risk Study Team, 2002), the prevalences of different types of victimisation among poly-victimised twins were similar to those reported in a non-twin study (Turner *et al.*, 2010), and no significant differences in mental health difficulties have been reported between twins and singletons (Kendler *et al.*, 1995).

### Implications

Our findings have practical implications. First, the promotive effect observed for the presence of a supportive adult suggests that early interventions aimed at increasing the availability of supportive figures in a child’s life or their perceptions of existing support may protect against the development of psychopathology in early adulthood. Interventions could, for example, focus on improving the relationship between children and their caregivers, ensuring schools have dedicated counsellors, enhancing the role and recognition of natural mentors (i.e., supportive adults outside the immediate family; Hurd and Zimmerman, 2014), and providing youth workers within offline and online communities. However, for such interventions to be effective, it will likely be important to provide children with the information and social skills required to access these potential sources of support and be able to build healthy interpersonal relationships (Bauer *et al.*, 2021). This underscores the importance of implementing interventions at multiple levels because individual-, family- and community-level factors interact with each other (García-Carrión *et al.*, 2019; Ungar and Theron, 2020). Second, the protective factors examined in our study were not specific to the population of poly-victimised children. Our findings therefore lend support to universally implemented interventions. However, these interventions can be costly, and the resources required for implementation are limited. We therefore recommend prioritising poly-victimised children first, given their higher levels of psychopathology compared to non-poly-victimised children, and prioritising individually targeted interventions over collective interventions. Furthermore, these interventions need to be evaluated before widespread implementation, taking into account unintended negative consequences associated with these prevention programmes, such as stigmatisation or increased stress (Durlak and Wells, 1998).

## Conclusion

Having at least one supportive adult was found to be associated with less psychopathology in early adulthood among both poly-victimised and non-poly-victimised children. Therefore, these findings suggest that mental health promotion strategies in youth should promote better availability and utilisation of supportive adults during childhood and be implemented universally. However, further research is needed with contemporary samples and in other contexts to replicate our findings. Moreover, future studies should investigate the effectiveness of interventions to prevent mental health difficulties that target poly-victimised children, given the higher burden of psychopathology that they experience in early adulthood.

## Supporting information

Blangis et al. supplementary material Blangis et al. supplementary material

## Data Availability

The dataset reported in the current article is not publicly available due to lack of informed consent and ethical approval for open access, but is available on request by qualified scientists. Requests require a concept paper describing the purpose of data access, ethical approval at the applicant’s institution, and provision for secure data access (for further details, see here: https://eriskstudy.com/data-access/). Supporting Stata code will become publicly available via F.B.’s GitHub account on publication: https://github.com/FloraBlangis.

## Supplementary material

The supplementary material for this article can be found at https://doi.org/10.1017/S2045796024000660.
