# Supplementary material for: Testing whether multi-level factors protect poly-victimised children against psychopathology in early adulthood: a longitudinal cohort study
Source: Epidemiol Psychiatr Sci. 2024 Nov 5;33:e58. doi: 10.1017/S2045796024000660 (PMC11561683; doi:10.1017/S2045796024000660)
Supplement: Blangis et al. supplementary material [file S2045796024000660sup001.docx]

**Supplementary Material**

Blangis F et al. Testing whether multi-level factors protect poly-victimised children against psychopathology in early adulthood: A longitudinal cohort study.

**Supplementary Text 1.** Additional details on the E-Risk Study sample

**Supplementary Text 2.** Assessment of victimisation in childhood

**Supplementary Text 3.** Description of dimensional measures of psychopathology within the E-Risk cohort at age 18

**Supplementary Figure 1. The structure of psychopathology at age 18 years in the E-Risk Cohort**

**Supplementary Table 1.** Associations between poly-victimisation and general psychopathology (main analysis), and associations between poly-victimisation and internalising, externalising and thought disorder dimensions, retrospective measure of poly-victimisation and general psychopathology, and only-severe poly-victimisation and general psychopathology and internalising, externalising and thought disorder dimensions (sensitivity analysis)

**Supplementary Table 2.** Associations between the presence of a supportive adult and internalising, externalising, and thought disorder dimensions among poly-victimised children

**Supplementary Table 3.** Associations between the presence of a supportive adult and general psychopathology among adults with retrospective report of victimisation

**Supplementary Table 4.** Associations between the presence of a supportive adult and internalising, externalising, and thought disorder dimensions among adults with retrospective report of victimisation

**Supplementary Table 5.** Associations between the presence of a supportive adult and general psychopathology among severe-only poly-victimised children

**Supplementary Table 6.** Associations between the presence of a supportive adult and internalising, externalising, and thought disorder dimensions among severe-only poly-victimised children

**Supplementary Text 1.** Additional details on the E-Risk Study sample

Participants were members of the Environmental Risk (E-Risk) Longitudinal Twin Study, which tracks the development of a nationally representative birth cohort of 2,232 British twin children. The sample was drawn from a larger birth register of twins born in England and Wales in 1994-1995 (Trouton *et al.*, 2002). Full details about the sample are reported elsewhere (Moffitt and E‐Risk Study Team, 2002). Briefly, the E-Risk sample was constructed in 1999-2000, when 1116 families (93% of those eligible) with same-sex 5-year-old twins participated in home-visit assessments. This sample comprised 56% monozygotic (MZ) and 44% dizygotic (DZ) twin pairs; sex was evenly distributed within zygosity (49% male). Families were recruited to represent the UK population of families with newborns in the 1990s, on the basis of residential location throughout England and Wales and mother’s age. Teenaged mothers with twins were over-selected to replace high-risk families who were selectively lost to the register through non-response. Older mothers having twins via assisted reproduction were under-selected to avoid an excess of well-educated older mothers.

Follow-up home-visits were conducted when children were aged 7, 10, 12 and 18 (participation rates were 98%, 96%, 96% and 93%, respectively). Home-visits at ages 5, 7, 10, and 12 years included assessments with participants as well as their mother (or primary caregiver); the home-visit at age 18 included interviews only with the participants. Each participant in a twin pair was assessed by a different interviewer. There were 2,066 E-Risk participants who were assessed at age 18. The average age of the participants at the time of the assessment was 18.4 years (SD = 0.36); all interviews were conducted after the 18th birthday. There were no differences between those who did and did not take part at age 18 in terms of socioeconomic status (SES) assessed when the cohort was initially defined (χ^2^ = 0.86, *p* = .65), age-5 IQ scores (t = 0.98, *p* = .33), age-5 internalising or externalising behaviour problems (t = 0.40, *p* = .69 and t = 0.41, *p* = .68, respectively), or childhood poly-victimisation (z=0.51, *p* = .61). The study sample represents the full range of socioeconomic conditions in Great Britain, as reflected in the families’ distribution on a neighbourhood-level socioeconomic index (ACORN [A Classification of Residential Neighbourhoods] developed by CACI Inc. for commercial use (CACI Information Services, 2006). E-Risk families ACORN distribution closely matches that of households nation-wide: 25.6% of E-Risk families live in “wealthy achiever” neighbourhoods compared to 25.3% of households nation-wide; 5.3% vs 11.6% live in “urban prosperity” neighbourhoods; 29.6% vs 26.9% live in “comfortably off” neighbourhoods; 13.4% vs 13.9% live in “moderate means” neighbourhoods; and 26.1% vs 20.7% live in “hard-pressed” neighbourhoods. E-Risk underrepresents urban prosperity neighbourhoods because such households are likely to be childless.

Parents gave informed consent and twins gave assent between 5-12 years and then informed consent at age 18. The Joint South London and Maudsley and the Institute of Psychiatry Research Ethics Committee approved each phase of the study.

**Supplementary Text 2.** Assessment of victimisation in childhood

We have previously reported evidence on the reliability and validity of our measurement of childhood victimisation (Danese *et al.*, 2017). Here we summarise the method. A team of interviewers visited each family at home when the twins reached ages 5, 7, 10 and 12 years. Each home-visit interview was guided by a series of questions in a booklet. Based on these interviews with the mothers, each interviewer coded in the booklet her initial impression of whether or not she thought a child had been maltreated. The interviewers also recorded notes about their experiences in the home, and if an interviewer was worried about a child, she met with the fieldwork coordinator to debrief. Sometimes, the Study had to make a referral to help a child. Codes, notes, and the fieldwork coordinator’s narratives from the debriefs have been saved over the years to create a dossier for each child with cumulative information about exposure to domestic violence between the mother and her partner; frequent bullying by peers; physical maltreatment by an adult; sexual abuse; emotional abuse and neglect; and physical neglect. All the component measures are outlined briefly below.

Physical domestic violence. Mothers reported about perpetration by and victimisation of 12 forms of physical violence (e.g., slapping, hitting, kicking, strangling) from the Conflict Tactics Scale (CTS) (Straus *et al.*, 1990), on three assessment occasions during the child’s first decade of life (when the children were 5, 7, and 10 years of age). Reports of either perpetration or victimisation constituted evidence of physical domestic violence. The CTS has between-partner inter-rater reliabilities of 0.76 for perpetration and 0.82 for victimisation (Magdol *et al.*, 1998). Families in which no physical violence took place were coded as 0 (55.2%); families in which physical violence took place on one occasion were coded as 1 (28.0%); and families in which physical violence took place on multiple occasions were coded as 2 (16.8%).

Bullying by peers. Experiences of victimisation by bullies were assessed using both mothers’ and children’s reports. During the interview, the following standard definition of bullying was read out: “Someone is being bullied when another child (a) says mean and hurtful things, makes fun, or calls a person mean and hurtful names; (b) completely ignores or excludes someone from their group of friends or leaves them out on purpose; (c) hits, kicks, or shoves a person, or locks them in a room; (d) tells lies or spreads rumours about them; and (e) other hurtful things like these. We call it bullying when these things happen often, and when it is difficult to make it stop. We do not call it bullying when it is done in a friendly or playful way.” Mothers were interviewed when children were 7, 10, and 12 years old and asked whether either twin had been bullied by another child, responding never, yes, or frequently. We combined mothers’ reports at child age 7 and 10 to derive a measure of victimisation during primary school. Mothers’ reports when the children were 12 years old indexed victimisation during secondary school. During private interviews with the children when they were 12 years old, the children indicated whether they had been bullied by another child during primary or secondary school. When a mother or a child reported victimisation, the interviewer asked them to describe what happened. Notes taken by the interviewers were later checked by an independent rater to verify that the events reported could be classified as instances of bullying operationally defined as evidence of (a) repeated harmful actions, (b) between children, and (c) where there is a power differential between the bully and the victim. Although inter-rater reliability between mothers and children was only modest (kappa = 0.20–0.29), reports of victimisation from both informants were similarly associated with children’s emotional and behavioural problems, suggesting that each informant provides a unique but meaningful perspective on bullying involvement (Shakoor *et al.*, 2011). We thus combined mother and child reports of victimisation to capture all instances of bullying victimisation for primary and secondary school separately: reported as not victimised by both mother and child; reported by either mother or child as being occasionally victimised; and reported as being occasionally victimised by both informants or as frequently victimised by either mother or child or both (Bowes *et al.*, 2013). We then combined these primary and secondary school ratings to create a bullying victimisation variable for the entire childhood period (5–12 years). Children who were never bullied in primary or secondary school or occasionally bullied during one of these time periods were coded as 0 (55.5%); children who were occasionally bullied during primary and secondary school, or frequently bullied during one of these time periods were coded as 1 (35.6%); and children who were frequently bullied at both primary and secondary school were coded as 2 (8.9%).

Physical and sexual harm by an adult. When the twins were aged 5, 7, 10 and 12, their mothers were interviewed about each twins’ experience of intentional harm by an adult. At age 5 we used the standardised clinical protocol from the MultiSite Child Development Project (Dodge *et al.*, 1990; Lansford *et al.*, 2002). At ages 7, 10, and 12 this interview was modified to expand its coverage of contexts for child harm. Interviews were designed to enhance mothers’ comfort with reporting valid child maltreatment information, while also meeting researchers’ responsibilities for referral under the U.K. Children Act. Specifically, mothers were asked whether either of their twins had been intentionally harmed (physically or sexually) by an adult or had contact with welfare agencies. If caregivers endorsed a question, research workers made extensive notes on what had happened, and indicated whether physical and/or psychological harm had occurred. Under the U.K. Children Act, our responsibility was to secure intervention if maltreatment was current and ongoing. Such intervention on behalf of E-Risk families was carried out with parental cooperation in all but one case. No families left the study following intervention. Over the years of data collection, the study developed a cumulative profile for each child, comprising the caregiver reports, recorded debriefings with research workers who had coded any indication of maltreatment at any of the successive home visits, recorded narratives of the successive caregiver interviews, and information from clinicians whenever the Study team made a child-protection referral. The profiles were reviewed at the end of the age–12 phase by two clinical psychologists. Inter-rater agreement between the coders was 90% for cases for whom maltreatment was identified (100% for cases of sexual abuse), and discrepantly coded cases were resolved by consensus review. These were coded as: 0 = no physical harm at any age; 1 = probable physical harm at any age; and 2 = definite physical harm at any age. There were 15.0% of children coded as probably being exposed to physical harm and 5.1% as definitely physically harmed by 12 years of age. There were 1.5% of the children coded as being exposed to sexual abuse.

Emotional abuse and neglect were coded from research workers’ narratives of the home visits at ages 5, 7, 10, and 12. We coded quite severe examples of parental behaviour observed. For example, a mother who had schizophrenia screamed and swore at the children throughout the home visit. As another example, a father who was drunk during the home visit repeatedly spoke abusively to the children in front of the research workers. We found that coders could not empirically separate emotional abuse and emotional neglect in a reliable way and thus such experiences were coded together as emotional abuse/neglect. Inter-rater agreement between the coders exceeded 85% for cases with emotional abuse and neglect, and discrepant cases were resolved by consensus review. Children with no evidence of emotional abuse/neglect were coded as 0 (88.3%), those where there was some indication of emotionally inappropriate/potentially abusive or neglectful behaviour were coded as 1 (8.7%), and where there was evidence of severe emotional abuse/neglect the children were coded as 2 (3.0%).

Physical neglect. The cumulative observations of the physical state of the home environment documented by the research workers during home visits to the twins at ages 5, 7, 10 and 12 were reviewed by two raters for evidence of physical neglect. This was defined as any sign that the caretaker was not providing a safe, sanitary, or healthy environment for the child. This included the child not having proper clothing or food, as well as grossly unsanitary home environments. (However, this did not include a family living in a deprived or crime-ridden neighbourhood). Inter-rater agreement between the coders was 85%, and discrepantly coded cases were resolved by consensus review. Children with no evidence of physical neglect were coded as 0 (90.9%), those for whom there was an indication of minor physical neglect were coded as 1 (7.1%), and where there was evidence of severe physical neglect the children were coded as 2 (2.0%).

Childhood poly-victimisation. Finkelhor *et al.* (2007) operationalise poly-victimisation as the total number of victimisation types that a child experiences. For the current analysis, we summed all of the different victimisation experiences that received a code of ‘1’ mild or ‘2’ severe for each child and then we dichotomised the poly-victimisation variable into those children who experienced two or more types of *mild or severe* victimisation before age 12 (N = 720, 35%) compared to one or none (N = 1,346, 65%).

Retrospective self-reports. Maltreatment was measured retrospectively using the Childhood Trauma Questionnaire when E-Risk participants were aged 18 (CTQ) (Bernstein and Fink, 1998). The CTQ is a 25-item questionnaire used for retrospective recall of five forms of maltreatment, and has high inter-rater reliability and construct and convergent validity (Fink *et al.*, 1995). The CTQ is also one of the most commonly used retrospective measures of childhood maltreatment, thus increasing the comparability of the present study with previous and future research. Participants reported on their personal experiences of physical, sexual and emotional abuse, and physical and emotional neglect for the period before they were 12 years old (i.e., before entering secondary school). Almost all (99.5%; N = 2,055) E-Risk participants who took part in the age-18 assessment completed the CTQ. Maltreatment scores were dichotomised following CTQ guidelines (Bernstein and Fink, 1998) to represent none/low (0) versus moderate/severe (1) maltreatment. To allow retrospective self-reports of maltreatment to be compared to prospective informant-reports, emotional abuse and emotional neglect were combined so that a moderate/severe score for emotional abuse and/or emotional neglect represented a moderate/severe score for ‘emotional abuse/neglect’. For comparability to the prospective measure of poly-victimisation, we added domestic violence and bullying by peers from the prospective report to the retrospectively “self-reported maltreatment” variable. As with the prospective reports, we created a poly-victimisation variable following the same procedure described above but using the retrospective self-reports of maltreatment.

**Supplementary Text 3.** Description of dimensional measures of psychopathology within the E-Risk cohort at age 18

*Assessment of symptoms of mental disorder*

At age 18, participants were assessed in private interviews about past-year symptoms of mental disorders (Schaefer *et al.*, 2018). Five externalising-spectrum disorder symptoms were assessed: Diagnostic and Statistical Manual of Mental Disorders 4^th^ edition (DSM–IV) (American Psychiatric Association, 1994) symptoms of alcohol dependence and cannabis dependence assessed via the Diagnostic Interview Schedule (DIS) (Robins *et al.*, 1995); conduct disorder assessed by inquiring about DSM–IV symptoms; symptoms of tobacco dependence assessed via the Fagerstrom Test for Nicotine Dependence (Heatherton *et al.*, 1991); and attention-deficit/ hyperactivity disorder (ADHD) assessed by inquiring about DSM 5^th^ edition (DSM–5) (American Psychiatric Association, 2013) symptoms (Agnew-Blais *et al.*, 2016). Four internalising-spectrum disorder symptoms were assessed: DSM–IV symptoms of depression, generalised anxiety disorder, and posttraumatic stress disorder (PTSD) assessed via the DIS (Robins *et al.*, 1995), and symptoms of eating disorder assessed via the SCOFF (Morgan *et al.*, 1999). Thought disorder symptoms were assessed in two ways: first, participants were asked 7 items about delusions and hallucinations (psychotic-like experiences: e.g., “Have other people ever read your thoughts?”; “Have you ever thought you were being followed or spied on?”; “Have you ever heard voices that other people cannot hear?”) (Polanczyk *et al.*, 2010). Second, participants were asked 6 items about unusual thoughts and feelings (prodromal symptoms: e.g., “My thinking is unusual or frightening”; “People or places I know seem different”), drawing on item pools since formalised in prodromal psychosis instruments, including the PRIME-screen and SIPS (Loewy *et al.*, 2011).

*The structure of psychopathology*

Using confirmatory factor analysis, two standard models (Brunner *et al.*, 2012; Rindskopf and Rose, 1988) that are frequently used to examine hierarchically structured constructs were estimated: a correlated-factors model with three factors (representing Internalising, Externalising, and Thought Disorder symptoms) and a bi-factor model specifying a General Psychopathology factor (**Supplementary Figure 1**) in addition to the three specific factors. Decisions about symptom-factor loadings were guided by the Hierarchical Taxonomy of Psychopathology consortium

(<https://medicine.stonybrookmedicine.edu/HITOP/AboutHiTOP>) (Kotov *et al.*, 2017). Symptoms corresponding to disorders of distress (depression, generalised anxiety disorder, and PTSD) and eating pathology loaded on the Internalising factor; symptoms corresponding to disorders of substance use (alcohol, cannabis, tobacco) and oppositional behaviour (conduct disorder) and ADHD loaded on the Externalising factor; and symptoms corresponding to disorders associated with psychosis loaded on the Thought Disorder factor. Confirmatory factor analyses were run as two-level clustered models to account for the nesting of twins within families, with analyses performed in MPlus v7.4 (Muthén, 2017) using the robust maximum likelihood estimator (MLR) to provide standard errors that are robust to non-normality and non-independence of observations.

Both models fit the data well as assessed by the Akaike Information Criterion (AIC), Bayesian Information Criterion (BIC) and the Sample Adjusted BIC, although the bi-factor model demonstrated marginally superior fit.

For the correlated-factors model, AIC = 42,987.116, BIC = 43,488.486, Sample Adjusted BIC = 43,205.726. Loadings on each of the three factors were all positive, generally high (all p’s < .001) and averaged 0.680 (Externalising: average loading = 0.638; Internalising: average loading = 0.654; Thought Disorder: average loading = 0.836). Correlations between the three factors were all positive and ranged from 0.552 between Externalising and Thought Disorder to 0.756 between Internalising and Thought Disorder. Thus, this model confirmed that three correlated factors (i.e., Internalising, Externalising, and Thought Disorder) explained the structure of the 11 symptom scales examined in the E-Risk twins at age 18.

For the bi-factor model, AIC = 42,897.350, BIC = 43,443.787, Sample Adjusted BIC = 43,135.609. Loadings on the General Psychopathology factor (“p”) were all positive, generally high (all p’s < .001) and averaged 0.519; the highest standardised loadings were for psychotic symptoms (0.759 and 0.592), major depressive episode (0.718), eating disorders (0.574), and generalised anxiety disorder (0.567). Similarly, the loadings for the three specific factors were all positive and averaged 0.507 for Externalising, 0.270 for Internalising, and 0.496 for Thought Disorder. Thus, this model confirmed that a bi-factor structure (i.e., with a General Psychopathology factor and three specific Internalising, Externalising, and Thought Disorder factors) explained the structure of the 11 symptom scales examined in the E-Risk twins at age 18.


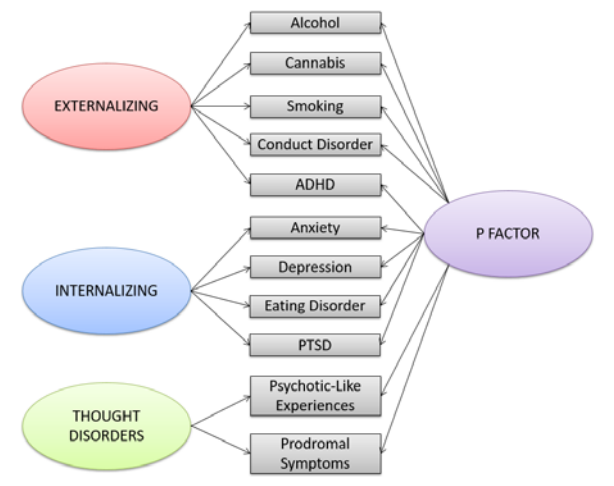
(A)

**(**B)


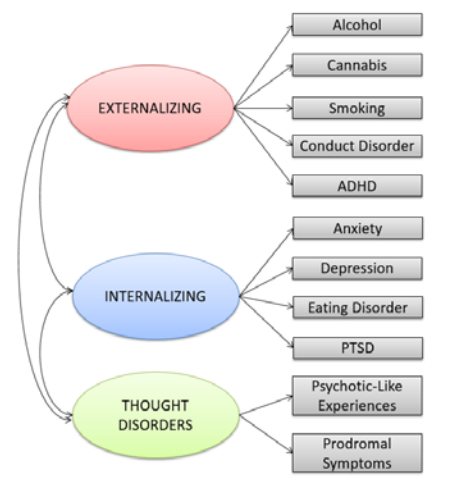


# **Supplementary Figure 1. The structure of psychopathology at age 18 years in the E-Risk Cohort**

*Note.* (A) Bi-factor model, (B) Correlated-factors model. Coloured ovals represent latent (unobserved) continuous symptom trait factors; grey boxes represent age-18 observed scores on symptom scales corresponding to each disorder. ADHD = attention-deficit/hyperactivity disorder, PTSD = post-traumatic stress disorder. P-Factor represents the factor of General Psychopathology. Figure reproduced from Schaefer *et al.* (2018).

**Supplementary Table 1.** Associations between poly-victimisation and general psychopathology (main analysis), and associations between poly-victimisation and internalising, externalising and thought disorder dimensions, retrospective measure of poly-victimisation and general psychopathology, and only-severe poly-victimisation and general psychopathology and internalising, externalising and thought disorder dimensions (sensitivity analysis)

|  |  | **General psychopathology** | **Internalising disorders** | **Externalising disorders** | **Thought disorders** |
| --- | --- | --- | --- | --- | --- |
|  |  | **β (95% CI)** | **β (95% CI)** | **β (95% CI)** | **β (95% CI)** |
| **Poly-victimisation (prospective measure)** | Unadjusted | **6.74 [5.19; 8.30]** | **6.25 [4.69; 7.80]** | **6.81 [5.22; 8.39]** | **6.37 [4.82; 7.93]** |
|  | Adjusted^a^ | **4.80 [3.13; 6.47]** | **4.63 [2.98; 6.27]** | **4.60 [2.88; 6.33]** | **4.45 [2.79; 6.12]** |
| **Poly-victimisation (retrospective measure)** | Unadjusted | **9.76 [8.07; 11.46]** | **9.32 [7.63; 11.00]** | **9.07 [7.36; 10.79]** | **9.20 [7.51; 10.90]** |
|  | Adjusted^a^ | **7.89 [6.11; 9.67]** | **7.70 [5.95; 9.46]** | **7.03 [5.20; 8.85]** | **7.38 [5.61; 9.15]** |
| **Only-severe**  **poly-victimisation**  **(prospective measure)** | Unadjusted | **10.21 [6.78; 13.63]** | **9.28 [5.81; 12.75]** | **11.76 [8.67; 14.84]** | **9.28 [5.76; 12.80]** |
|  | Adjusted^a^ | **7.35 [3.96; 10.74]** | **6.84 [3.48; 10.21]** | **8.63 [5.39; 11.86]** | **6.48 [3.01; 9.95]** |
| ^a^Adjusted for biological sex, family socioeconomic status, and family history of psychiatric disorders.  CI, confidence interval.  All analyses account for the non-independence of twin observations.  Bold text indicates 95% CIs that do not include zero. | | | | | |

**Supplementary Table 2.** Associations between the presence of a supportive adult and internalising, externalising, and thought disorder dimensions among poly-victimised children

|  |  | **Internalising disorders** | | **Externalising disorders** | | **Thought disorders** | |  |  |
| --- | --- | --- | --- | --- | --- | --- | --- | --- | --- |
| **Protective factor** |  | **β (95% CI)** | **Standardised β adjusted^a^** | **β (95% CI)** | **Standardised β adjusted^a^** | **β (95% CI)** | **Standardised β adjusted^a^** | |  |
| **Supportive adult** | Unadjusted | **-0.55 [-0.93; -0.17]** | - | **-0.60 [-0.92; -0.28]** | - | **-0.67 [-1.05; -0.30]** | - | |  |
|  | Adjusted^a^ | **-0.55 [-0.93; -0.17]** | -0.13 | **-0.50 [-0.84; -0.16]** | -0.12 | **-0.62 [-1.01; -0.23]** | -0.15 | |  |
| ^a^Adjusted for biological sex, family socioeconomic status, and family history of psychiatric disorders.  CI, confidence interval.  All analyses account for the non-independence of twin observations.  The n varied from n=693 to n=698, due to different levels of completion of the measures.  Bold text indicates 95% CIs that do not include zero. | | | | | | | | | |

**Supplementary Table 3.** Associations between the presence of a supportive adult and general psychopathology among adults with retrospective report of victimisation

|  | **General psychopathology** | | |
| --- | --- | --- | --- |
| **Protective factor** | **β (95% CI) Unadjusted** | **β (95% CI) Adjusted^a^** | **Standardised β adjusted^a^** |
| **Supportive adult** | **-0.78 [-1.19; -0.37]** | **-0.77 [-1.19; -0.35]** | -0.18 |
| ^a^Adjusted for biological sex, family socioeconomic status, and family history of psychiatric disorders.  CI, confidence interval.  All analyses account for the non-independence of twin observations.  The n varied from n=527 to n=532, due to different levels of completion of the measures.  Bold text indicates 95% CIs that do not include zero. | | | |

**Supplementary Table 4.** Associations between the presence of a supportive adult and internalising, externalising, and thought disorder dimensions among adults with retrospective report of victimisation

|  |  | **Internalising disorders** | | **Externalising disorders** | | **Thought disorders** | |
| --- | --- | --- | --- | --- | --- | --- | --- |
| **Protective factor** |  | **β (95% CI)** | **Standardised β adjusted^a^** | **β (95% CI)** | **Standardised β adjusted^a^** | **β (95% CI)** | **Standardised β adjusted^a^** |
| **Supportive adult** | Unadjusted | **-0.68 [-1.11; -0.25]** | **-** | **-0.73 [-1.07; -0.39]** | - | -**0.79 [-1.21; -0.36]** |  |
|  | Adjusted^a^ | **-0.72 [-1.14; -0.29]** | -0.17 | **-0.66 [-1.02; -0.30]** | -0.16 | **-0.76 [-1.19; -0.32]** | -0.18 |
| ^a^Adjusted for biological sex, family socioeconomic status, and family history of psychiatric disorders.  CI, confidence interval.  All analyses account for the non-independence of twin observations.  The n varied from n=527 to n=532, due to different levels of completion of the measures.  Bold text indicates 95% CIs that do not include zero. | | | | | | | |

**Supplementary Table 5.** Associations between the presence of a supportive adult and general psychopathology among severe-only poly-victimised children

|  | **General psychopathology** | | |
| --- | --- | --- | --- |
| **Protective factor** | **β (95% CI) unadjusted** | **β (95% CI) adjusted^a^** | **Standardised β adjusted^a^** |
| **Supportive adult** | -0.57 [-1.32; 0.18] | -0.55 [-1.24; 0.13] | -0.16 |
| ^a^Adjusted for biological sex, family socioeconomic status, and family history of psychiatric disorders.  CI, confidence interval.  All analyses account for the non-independence of twin observations.  The n varied from n=124 to n=125, due to different levels of completion of the measures. | | | |

**Supplementary Table 6.** Associations between the presence of a supportive adult and internalising, externalising, and thought disorder dimensions among severe-only poly-victimised children

|  |  | **Internalising disorders** | | **Externalising disorders** | | **Thought disorders** | |
| --- | --- | --- | --- | --- | --- | --- | --- |
| **Protective factor** |  | **β (95% CI)** | **Standardised β adjusted^a^** | **β (95% CI)** | **Standardised β adjusted^a^** | **β (95% CI)** | **Standardised β adjusted^a^** |
| **Supportive adult** | Unadjusted | -0.47 [-1.19; 0.26] | - | -0.29 [-0.83; 0.25] | - | -0.71 [-1.53; 0.11] | - |
|  | Adjusted^a^ | -0.46 [-1.10; 0.19] | -0.13 | -0.22 [-0.78; 0.34] | -0.07 | -0.71 [-1.46; 0.05] | -0.19 |
| ^a^Adjusted for biological sex, family socioeconomic status, and family history of psychiatric disorders.  CI, confidence interval.  All analyses account for the non-independence of twin observations.  The n varied from n=124 to n=125, due to different levels of completion of the measures. | | | | | | | |

**Supplementary references**

**Agnew-Blais JC, Polanczyk GV, Danese A, Wertz J, Moffitt TE and Arseneault L** (2016) Evaluation of the persistence, remission, and emergence of attention-deficit/hyperactivity disorder in young adulthood. *JAMA Psychiatry* **73**, 713-720.

**American Psychiatric Association** (1994) Diagnostic and Statistical Manual of Mental Disorders. 4th ed. American Psychiatric Association: Washington, DC.

**American Psychiatric Association** (2013) Diagnostic and Statistical Manual of Mental Disorders. 5th ed. American Psychiatric Association: Washington, DC.

**Bernstein DP and Fink L** (1998) Manual for the Childhood Trauma Questionnaire. Psychological Corporation: New York.

**Bowes L, Maughan B, Ball H, Shakoor S, Ouellet-Morin I, Caspi A, Moffitt TE and Arseneault L** (2013) Chronic bullying victimization across school transitions: the role of genetic and environmental influences. *Development and Psychopathology* **25**, 333-346.

**Brunner M, Nagy G and Wilhelm O** (2012) A tutorial on hierarchically structured constructs. *Journal of Personality* **80**, 796-846.

**CACI Information Services** (2006) ACORN user guide. CACI: London, UK.

**Danese A, Moffitt TE, Arseneault L, Bleiberg BA, Dinardo PB, Gandelman SB, Houts R, Ambler A, Fisher HL, Poulton R and Caspi A** (2017) The origins of cognitive deficits in victimized children: implications for neuroscientists and clinicians. *American Journal of Psychiatry* **174**, 349-361.

**Dodge KA, Bates JE and Pettit GS** (1990) Mechanisms in the cycle of violence. *Science* **250**, 1678-1683.

**Fink LA, Bernstein D, Handelsman L, Foote J and Lovejoy M** (1995) Initial reliability and validity of the childhood trauma interview: a new multidimensional measure of childhood interpersonal trauma. *American Journal of Psychiatry* **152**, 1329-1335.

**Finkelhor D, Ormrod RK and Turner HA** (2007) Poly-victimization: a neglected component in child victimization. *Child Abuse and Neglect* **31**, 7-26.

**Heatherton TF, Kozlowski LT, Frecker RC and Fagerström KO** (1991) The Fagerström Test for Nicotine Dependence: a revision of the Fagerström Tolerance Questionnaire. *British Journal of Addiction* **86**, 1119-1127.

**Kotov R, Krueger RF, Watson D, Achenbach TM, Althoff RR, Bagby RM, Brown TA, Carpenter WT, Caspi A, Clark LA, Eaton NR, Forbes MK, Forbush KT, Goldberg D, Hasin D, Hyman SE, Ivanova MY, Lynam DR, Markon K, Miller JD, Moffitt TE, Morey LC, Mullins-Sweatt SN, Ormel J, Patrick CJ, Regier DA, Rescorla L, Ruggero CJ, Samuel DB, Sellbom M, Simms LJ, Skodol AE, Slade T, South SC, Tackett JL, Waldman ID, Waszczuk MA, Widiger TA, Wright AGC and Zimmerman M** (2017) The Hierarchical Taxonomy of Psychopathology (HiTOP): A dimensional alternative to traditional nosologies. *Journal of Abnormal Psychology* **126**, 454-477.

**Lansford JE, Dodge KA, Pettit GS, Bates JE, Crozier J and Kaplow J** (2002) A 12-year prospective study of the long-term effects of early child physical maltreatment on psychological, behavioral, and academic problems in adolescence. *Archives of Pediatrics and Adolescent Medicine* **156**, 824-830.

**Loewy RL, Pearson R, Vinogradov S, Bearden CE and Cannon TD** (2011) Psychosis risk screening with the Prodromal Questionnaire--brief version (PQ-B). *Schizophrenia Research* **129**, 42-46.

**Magdol L, Moffitt TE, Caspi A and Silva PA** (1998) Developmental antecedents of partner abuse: a prospective-longitudinal study. *Journal of Abnormal Psychology* **107**, 375.

**Moffitt TE and E‐Risk Study Team** (2002) Teen-aged mothers in contemporary Britain. *Journal of Child Psychology and Psychiatry* **43**, 727-742.

**Morgan JF, Reid F and Lacey JH** (1999) The SCOFF questionnaire: assessment of a new screening tool for eating disorders. *British Medical Journal* **319**, 1467-1468.

**Muthén L** (2017) Mplus: Statistical Analysis with Latent Variables: User's Guide (Version 8). Muthén & Muthén: Los Angeles.

**Polanczyk G, Moffitt TE, Arseneault L, Cannon M, Ambler A, Keefe RS, Houts R, Odgers CL and Caspi A** (2010) Etiological and clinical features of childhood psychotic symptoms: results from a birth cohort. *Archives of General Psychiatry* **67**, 328-338.

**Rindskopf D and Rose T** (1988) Some theory and applications of Confirmatory Second-Order Factor Analysis. *Multivariate Behavioral Research* **23**, 51-67.

**Robins L, Cottler L, Bucholz K, Compton W, North C and Rourke K** (1995) Diagnostic Interview Schedule for DSM-IV. Washington University Press: St Louis.

**Schaefer JD, Moffitt TE, Arseneault L, Danese A, Fisher HL, Houts R, Sheridan MA, Wertz J and Caspi A** (2018) Adolescent victimization and early-adult psychopathology: approaching causal inference using a longitudinal twin study to rule out noncausal explanations. *Clinical Psychological Science* **6**, 352-371.

**Shakoor S, Jaffee SR, Andreou P, Bowes L, Ambler AP, Caspi A, Moffitt TE and Arseneault L** (2011) Mothers and children as informants of bullying victimization: results from an epidemiological cohort of children. *Journal of Abnormal Child Psychology* **39**, 379-387.

**Straus MA, Gelles RJ and Asplund LM** (1990) Measuring intrafamily conflict and violence: The Conflict Tactics (CT) scales*.* In *Physical violence in American families: Risk factors and adaptations to violence in 8,145 families* (Ed. MA Straus and RG Gelles), pp. 403–424. Transaction Press: New Brunswick.

**Trouton A, Spinath FM and Plomin R** (2002) Twins early development study (TEDS): a multivariate, longitudinal genetic investigation of language, cognition and behavior problems in childhood. *Twin Research* **5**, 444-448.
